# A rowing-specific mindfulness intervention: Effects on mindfulness, flow, reinvestment, and performance

**DOI:** 10.3389/fpsyg.2022.871804

**Published:** 2022-09-07

**Authors:** Katherine V. Sparks, Christopher Ring

**Affiliations:** School of Sport, Exercise and Rehabilitation Sciences, University of Birmingham, Birmingham, United Kingdom

**Keywords:** intervention, flow, mindfulness, rowing, reinvestment

## Abstract

Mindfulness can benefit athletes’ mindset and performance. These benefits may be enhanced by sport-specific mindfulness interventions. Accordingly, our objectives were 2-fold: first, to develop a rowing-specific mindfulness intervention, and second, to investigate its effects on mindfulness, flow, reinvestment, and rowing performance. Rowers were randomly assigned to either a 6-week rowing-specific mindfulness intervention (*n* = 23), which included generic and rowing-specific practices, or a control group (*n* = 21). Rowers completed pre-test and post-test measures of performance, mindfulness, flow, and rowing-specific reinvestment. Lastly, rowers completed an evaluation form following the intervention. The results demonstrated that the intervention group increased flow, mindfulness, and improved performance, additionally conscious motor processing decreased from pre-test to post-test. However, the intervention did not preferentially change mindfulness or reinvestment compared to control. Participants provided favorable feedback and evaluated the intervention positively. Our 6-week rowing-specific mindfulness intervention promoted flow, encouraged mindfulness, and aided performance. Thus, we provide preliminary explorative evidence that a sport-specific mindfulness intervention can benefit athletes. We recommend that future research, with large sample sizes and improved home practice, should examine mediators and moderators of the mindfulness-performance relationship.

## Introduction

Coaches and sport psychologists are continuously seeking methods to help athletes fulfill their potential in training and competition. These methods, which include imagery, pre-performance routines, and self-talk, have mixed performance benefits (Brown and Fletcher, 2017; Gröpel and Mesagno, 2019). Mindfulness, a practice which emphasizes open non-judgmental awareness and moment-to-moment attention (Vidic and Cherup, 2021), is an increasingly popular new method. Indeed, mindfulness should allow athletes to accept their current experience and keep their focus on the present moment, which could facilitate peak performance and attenuate processes linked to choking, such as reinvestment (Sparks et al., 2021b). Building on this cross-sectional research, the current study employed an experimental design to evaluate the effects of a sport-specific mindfulness intervention on competitive rowers’ thoughts and actions.

Mindfulness is a non-judgmental moment-to-moment awareness of current experiences, thoughts, and emotions (Kabat-Zinn, 1982). It has been described as both a trait and state. Trait mindfulness is the tendency to exhibit non-judgmental present moment awareness, while state mindfulness is to be mindful in the present moment. Mindfulness-based interventions have been developed to cultivate mindfulness, and they tend to include practices, such as guided meditations, body scans, gentle stretching, and group discussions (Creswell et al., 2019). These interventions were first employed in the therapeutic setting, such as for chronic injuries, mental and physical illnesses, and have demonstrated success in aiding recovery or reducing symptoms (Eberth and Sedlmeier, 2012; Rycroft-Malone et al., 2017). Despite this success, it took three decades for mindfulness-based interventions to be introduced to sport. Mindfulness was proposed to enhance performance through processes that contrast with traditional psychological skills training: the former promotes acceptance and non-judgment, while the latter teaches athletes to control or suppress thoughts (Birrer et al., 2012). Mindful athletes are more accepting of their current experiences, thoughts, and emotions, and therefore do not attempt to control or suppress their internal processes which can increase rumination or frequency of unwanted thoughts (Gardner and Moore, 2004; Vidic and Cherup, 2021). Furthermore, mindfulness can increase present moment self-awareness, which should allow athletes to banish thoughts related to post or future events, and instead focus on relevant present performance cues (Gardner and Moore, 2004). These mindful processes should allow them to adapt their movements to the everchanging competitive environment (Toner et al., 2015).

A number of frequently used mindfulness interventions for sport have been developed, including: Mindfulness-Acceptance-Commitment (Gardner and Moore, 2004), Mindful Sport Performance Enhancement Training (Kaufman et al., 2009), and Mindfulness Meditation Training in Sport (Baltzell and Summers, 2018). These interventions have had mixed success in enhancing performance or impacting performance-relevant outcomes, such as flow, anxiety, focus, and self-confidence (Bühlmayer et al., 2017; Noetel et al., 2017; Corbally et al., 2020). Some of these studies have documented increases in flow (Kee and Wang, 2008; Scott-Hamilton et al., 2016) and performance (Solberg et al., 1996; Thompson et al., 2011; Zhang et al., 2016), whereas others have not (De Petrillo et al., 2009; Wolanin and Schwanhausser, 2010; Aherne et al., 2011; Pineau et al., 2014). This mixed evidence could be due to methodological weakness, such as lack of randomization, blinding, and/or controls (Noetel et al., 2017). Importantly, the mechanism underlying the beneficial effects of mindfulness on performance has yet to be established, though several candidate mechanisms proposed.

One of the possible mechanisms, that have been investigated, is that mindfulness training facilitates flow (Kaufman et al., 2009; Thompson et al., 2011; Cathcart et al., 2014; Hill et al., 2020). Flow is an intrinsically rewarding and harmonious psychological state involving intense focus and absorption in a specific activity where someone perceives balance between their ability and the task demands (Csikszentmihalyi and Csikzentmihaly, 1990). Flow comprises nine dimensions: challenge-skill balance, action-awareness emerging, sense of control, clear goals, concentration on the task, unambiguous feedback, transformation of time, autotelic experience, and loss of self-consciousness (Csikszentmihalyi and Csikzentmihaly, 1990). Flow is not something that can be taught, and, therefore, being able to increase the likelihood of it occurring is considered beneficial for athletes (Baltzell and Summers, 2018). The flow experience shares similarities with mindfulness; both concern purposeful, present-moment awareness. When in a flow-state, the athlete is fully focused on the present, and no external or internal distractions occupy the mental space – this is like being mindful (Jackson, 2016). Therefore, researchers have proposed that teaching athletes to be more mindful should increase their likelihood to be in flow and thereby help them to reach their peak performance (Landhäußer and Keller, 2012).

Mindfulness may also improve an athlete’s selective attention (van den Hurk et al., 2010) and attention flexibility (Hodgins and Adair, 2010). Consequently, it has been proposed that mindfulness may regulate athletes’ self-focus and thereby attenuate or mitigate reinvestment (Birrer et al., 2012; Josefsson et al., 2017; Shaabani et al., 2020). Reinvestment describes the situation where individuals consciously control and monitor their motor processes leading to a breakdown in the automaticity of skill execution (Masters, 1992; Masters et al., 1993; Masters and Maxwell, 2008). Reinvestment has also been described as a trait, measured using the Reinvestment Scale (Masters et al., 1993) and Movement-specific Reinvestment Scale (Masters et al., 2005). Reinvestment comprises conscious motor processing (CMP), or awareness about the control of movement, and movement-specific self-consciousness (MSC), or monitoring of and public concern of movement style.

Research has demonstrated that reinvestment is associated with poor performance of pressurized laboratory-based sport skills (e.g., Chell et al., 2003; Jackson et al., 2006; Zhu et al., 2011; Orn, 2017). Nevertheless, such studies cannot readily generalize to sport competition (Toner and Moran, 2021), where pressure is more potent (Masters and Maxwell, 2008). Nonetheless, some field studies have also revealed that reinvestment is associated with poor performance (Iwatsuki and Wright, 2016; Sparks et al., 2021a,b). Importantly, field studies revealed that MSC and not CMP was related to rowing performance during regatta races, which, with crowds watching on the riverbank and coaches filming, probably evoked self-consciousness of the rowing stroke (Sparks et al., 2021a,b). Similarly, MSC but not CMP predicted disruption to top-spin and backhand table-tennis shots in the presence of an audience and camera (Rad et al., 2022) Consequently, CMP and MSC components of reinvestment are worth examined separately. Nevertheless, in general, reinvestment can have a detrimental impact on performance and, therefore, it is important for athletes to find ways to prevent conscious motor processing and movement self-consciousness.

Mindfulness is a good candidate because it allows performers to accept an unexpectedly poor performance instead of ruminating over it or trying to consciously control movements to prevent it from happening again (Birrer et al., 2012). Additionally, present moment awareness prevents rumination of experiences, thoughts, or emotions, and thereby helps athletes perform autonomously (Birrer et al., 2012; Röthlin et al., 2016; Josefsson et al., 2017). Sparks et al. (2021b) found that dispositional mindfulness attenuated the potentiating effect of reinvestment on the anxiety-performance relationship in rowers (Sparks et al., 2021b). This correlational evidence shows that mindfulness can aid performance. Clearly, the moderating effect of mindfulness warrants further investigation using experimental study designs.

Despite evidence that mindfulness-based interventions have a positive impact on performance, they typically last 6–12 weeks, with an average of 14 sessions. However, athletes with demanding schedules may not be able to engage with large numbers of sessions (Bühlmayer et al., 2017; Baltzell and Summers, 2018). Consequently, shorter interventions may be more convenient. Additionally, athletes and coaches may be skeptical about the use and benefits of mindfulness in sport (Vidic et al., 2018). Such skepticism may arise from poor understanding of mindfulness, especially with most intervention being generic rather than sport specific. Therefore, developing a sport-specific mindfulness intervention should improve an athlete’s understanding of mindfulness and make it easier for them to apply mindfulness to their sport (Hopper, 2017).

Scott-Hamilton et al. (2016) conducted an 8-week mindfulness intervention for competitive cyclists and incorporated mindful spinning into the training. The intervention increased both mindfulness and flow compared to control. These beneficial effects were attributed to the inclusion of sport-specific practice; with the cyclists able to apply the mindfulness processes to their sport. However, it is worth noting that two popular interventions—Mindful Sport Performance Enhancement (MSPE) and Mindfulness Meditation Training in Sport (MMTS)—included sport-specific scripts for only one of eight sessions (Baltzell and Summers, 2018). Therefore, a mindfulness-based intervention that is saturated with sport-specific practices may be expected to be more effective at cultivating mindfulness. It is also possible that a sport-specific intervention may require fewer sessions than a generic intervention.

The present study developed and evaluated a multi-session rowing-specific mindfulness intervention and investigated its effects on mindfulness, flow, reinvestment, and rowing performance. In line with the abovementioned research, it was hypothesized that the intervention would increase mindfulness and flow (Cathcart et al., 2014; Scott-Hamilton et al., 2016), decrease reinvestment (Sparks et al., 2021b), and improve performance (Jones et al., 2020), compared to control.

## Materials and methods

### Participants and design

Our study adopted a cluster-randomized pre-test to post-test control intervention design. Participants were randomly allocated by boat club (i.e., a cluster), based on computer generated numbers, to either the mindfulness intervention or control. This arrangement avoided treatment contamination and facilitated a friendly environment to encourage group discussion. Forty-four rowers (32 females, 12 males) completed the study.

**Figure 1:**
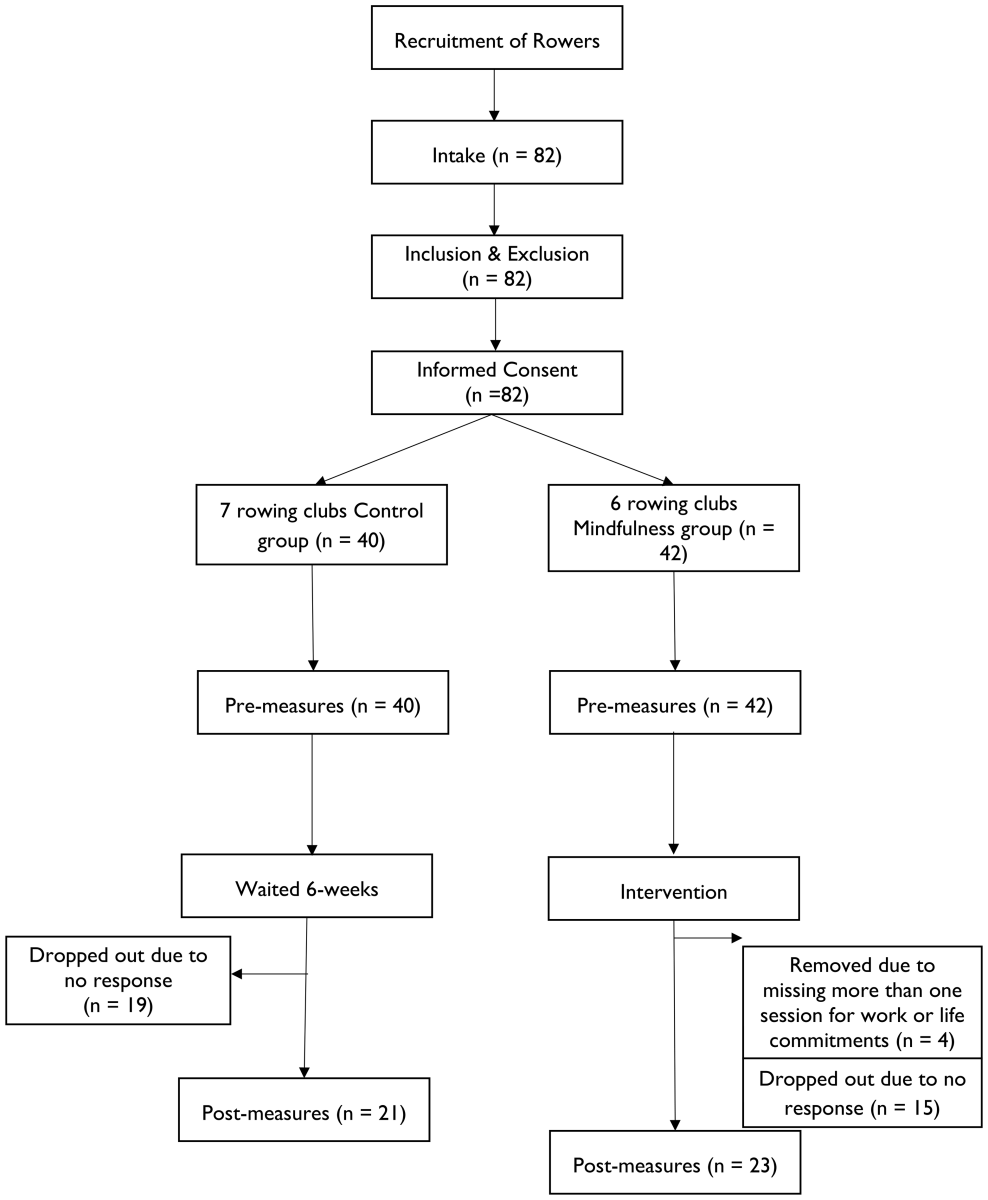
Participant recruitment and study flow.

Rowers were recruited through Twitter and Facebook using a recruitment poster. Additionally, recruitment emails to club secretaries in the United Kingdom were also sent out, this included the recruitment poster and details of the study in terms of eligibility, time commitment, and study involvement. Participants were only included if they had competed and had at least 1 year’s experience of rowing competitively and were aged 18 and over. A CONSORT study flow diagram is shown in Figure 1. All participants provided informed consent prior to partaking in the study. The study was approved by the University Ethics Committee.

### Procedure

Once rowers had agreed to take part in the intervention, they gave informed consent, and were randomly allocated by cluster (i.e., boat club) to either the mindfulness intervention (*n* = 23) or control (*n* = 21) groups. Rowers then completed the background questionnaire and all pre-test measures (see below). The mindfulness teacher then discussed with the captain/coach of the intervention group a suitable time for mindfulness training each week to ensure every rower could participate. The control group carried on with their daily routine for 6 weeks. Following the 6 weeks rowers completed post-test measures of performance. To maintain anonymity each rower was given an identification number for their pre-test and post-test questionnaire. Nevertheless, there was no blinding of the mindfulness practitioner (who also collected the data and ran the analyses), and, therefore there was potential for bias (Karanicolas et al., 2010). Finally, the fidelity of the intervention was measured using a self-reported measure of the number of minutes spent completing the home practice.

### Demographic questionnaire

The demographic questionnaire included questions regarding age, their boat club name, the type of boat they typically compete in (single, double/pair, quad/four, and eight), highest level they have competed at and any experience mindfulness.

### Rowing performance

We asked athletes one question about their training performance using a single item (“During the last 2 weeks I rate my training performance as…”) on a 10-point Likert scale, ranging from “1 = very poor” to “10 = very good” (Josefsson et al., 2019; Hasker, 2010).

#### Sport rating form

We also measured self-reported satisfaction with athletic performance (Hasker, 2010). Athletes were asked to rate their satisfaction on overall performance and 10 different dimensions that assessed physical (strength, endurance, quickness, fitness, and mechanics) and mental (concentration, motivation, and aggression). Each item was rated on a Likert scale of 1 (Very poor) to 5 (Very good). The scale has demonstrated good validity and reliability with alpha coefficients above 0.70 in previous research (Hasker, 2010) and the current study (Table 1).

**Table 1:** Demographic characteristics.

	Mindfulness (*n* = 23)	Control (*n* = 21)
Age (years) [Mean (*SD*)]	36.35 (20.4)	55.29 (12.37)
**Gender**		
Male	8	4
Female	15	17
Years spent Rowing [Mean (*SD*)]	10.39 (13.83)	16.81 (14.03)
**Highest competed level**		
Local	7	6
Regional	4	3
National	10	7
International	2	5
**Boat type**		
Single	2	2
Pair/Double	8	4
Four/Quad	7	9
Eight	6	6
**Mindfulness experience**		
Yes	15	15
No	8	6

### Rowing-specific reinvestment scale

A sport-specific version of the Movement Specific Reinvestment Scale (MSRS; Masters et al., 2005) was used to measure the conscious processes of rowing movements (Sparks et al., 2021a). The scale comprises the six-item Rowing-Specific Conscious Motor Processing (RS-CMP) subscale (e.g., “I paid attention to how I carried out my rowing movements”), and the six-item Rowing-Specific Movement Self-Consciousness (RS-MSC) subscale (e.g., “I believed that everyone was just looking at me and scrutinising my rowing”). Athletes rated their level of agreement on a seven-point Likert scale, anchored from 1 (strongly disagree) to 7 (strongly agree). The mean of the items for each subscale were calculated to measure RS-CMP and RS-MSC. The scale has demonstrated good validity (Sparks et al., 2021a) and alpha coefficient scores above 0.70 (Table 1).

### Sport-specific mindfulness

The Mindfulness Inventory for Sport (MIS) scale (Thienot et al., 2014) was used to assess the ability to be mindful in the context of sport. The MIS consists of three five-item subscales, measuring mindful awareness (e.g., “I am able to notice the sensations of excitement in my body”), non-judgmental thinking (e.g., “When I become aware that I am really upset because I am losing, I criticize myself for reacting this way”), and refocusing (e.g., “When I become aware that I am tense, I am able to quickly bring my attention back to what I should focus on”). Participants were asked to indicate how much each statement was generally reflective of what they experience when they row, on a scale of 1 (not at all) to 7 (very much). The non-judgmental subscale items were reverse scored. The mean of the items for each subscale were calculated to measure Mindful Awareness, Mindful Non-judgement, and Mindful Refocus. The scale has demonstrated good validity and reliability with alpha coefficients above 0.70 for each subscale (Table 1; Thienot et al., 2014).

### Dispositional flow

The short dispositional Core flow 10-item scale was developed following qualitative interviews with athletes (Jackson, 1996). The scale measures the subjective optimal experience of flow (Martin and Jackson, 2008), rather than the nine dimensions that lead to flow (Jackson and Eklund, 2002). The items include “I am totally involved,” “I am in the groove,” and “I feel in control.” Athletes rated the frequency that they experienced flow when they rowed on a Likert Scale, with anchors of 1 (Never) to 5 (Always). The scale presents good validity (Martin and Jackson, 2008) and good reliability with an alpha coefficient above 0.70 (Table 1).

### Program evaluation

Participants who completed the mindfulness intervention answered the Program Evaluation Questionnaire (PEQ; Minkler et al., 2020). We only included part of the PEQ regarding the five statements that assessed an athletes’ perception of program success. Athletes were asked, on a scale of 1 (Not at all helpful) to 7 (Extremely helpful), how helpful they found the mindfulness session in their “ability to be in the zone,” “ability to reduce anxiety,” “ability to focus,” “ability to let things go,” and “ability to be aware of and cope with feelings.” This scale has demonstrated good reliability with an alpha coefficient score above 0.70 (Table 1). We also included the question related to how confident the athlete felt incorporating mindfulness when rowing, which was rated between 1 (Not confident at all) and 7 (Extremely confident).

### Self-reported mindfulness completed

Participants who completed the mindfulness intervention also reported how many minutes they completed each week on total.

### Rowing-specific mindfulness intervention procedure

The mindfulness intervention was conducted by a facilitator who was a mindfulness teacher. The facilitator was a doctoral student in sport psychology. The facilitator possessed a mindfulness teacher qualification and practiced mindfulness for 6 months before the start of the intervention. This ensured that the facilitator had both knowledge and regular self-practice of mindfulness (Crane et al., 2012). The mindfulness sessions took place *via* videoconference (Zoom) due to COVID-19. Each session started with a 3–5-min mindful breathing meditation to bring everyone together and to create a mindful mindset for the session. This was followed by a debrief of the week’s home practice, any problems or issues with the practice, and advice for overcoming any barriers that were preventing completion of home practice were aired. This allowed us to monitor adherence to and identity ways to improve the home practice. The aims for the session were then introduced before the core session began. Participants could ask questions throughout the sessions. Discussion was also scheduled after each practice regarding how it could be implemented in their training or competition. Using a shared Google Drive, athletes received recordings of the practices that had been performed in the session and any erg or water-specific alternatives as these could not be performed during the online sessions (Supplementary material).

The rowing-specific mindfulness intervention was adapted from the MMTS and MSPE (Baltzell and Summers, 2018). It consisted of six sessions, 1 h per week for 6 weeks, with participants told to practice 10 min every day (Aherne et al., 2011). The sessions were as follows.

#### Week 1: Introduction to mindfulness

This covered general and sport-specific mindfulness, it included a mindfulness definition, theoretical understanding of mindfulness and rationale for it to be used in sport. Practice activities following this were the “chocolate” mindful exercise, a 3-min breathing exercise, and a 5-min brief rowing-specific centering exercise that was to be used before an erg(ometer) or water session.

#### Week 2: Importance of acceptance

This included further discussion around the core performance facilitators for mindfulness, which were taken and adapted from the MSPE. Participants were then taught about awareness, acceptance, clarity, decentering, and labeling of thoughts, feelings, and emotions in relation to rowing and how this could possibly impact sport performance. This session was based on the MMTS 2.0 session 2a and 2b.

#### Week 3: Understanding body awareness

We introduced body awareness as a means of entering the present moment. The body scan was first introduced but in the home practice a sport-specific body scan was introduced to be used on the erg or boat. Participants were also taught “soles of our feet” walking meditation, included was a rowing-specific alternative to “soles of our feet” for the rowing boat and erg.

#### Week 4: Self-compassion and dealing with negative mind-states

First, athletes were taught about the importance of self-compassion, followed by a practice where they directed their attention to wishing themselves and then their teammates well (see Module 4 in the MMTS, Baltzell and Summers, 2018). Following this, participants discussed the use of self-compassion and created their own self-compassion mantra. Participants then practised the acceptance of negative mind states specific to a personalised rowing scenario, were asked to label their state of mind, emotion or thought, and then practiced accepting it non-judgementally using a self-compassion phrase.

#### Week 5: Value-driven performance

First, there was a discussion around self-regulation and the problem with goals when under stress (see Module 5A MMTS, Baltzell and Summers, 2018). Following this, participants were taught about inspirational sport values and how they could develop their own. Athletes then listened to a values script to help them to recognize and establish their own values for performance. Participants then practiced implementing their inspirational sport values, self-compassion, and labeling in a rowing-specific race imagery activity to prevent rumination and negative thinking and to promote staying in the present.

#### Week 6: Importance of open awareness for refocus

The importance of an underlying broad awareness was first discussed before participants completed an open awareness script for sport. Participants were then taught about the need for a novelty state of mind when it comes to performance so they can be aware of the everchanging environment (Module 6, MMTS, Baltzell and Summers, 2018). Following this all athletes completed a brief pre-race mindfulness rowing practice for preparing to engage in the sport and were also introduced to the Focus Circle (Henriksen et al., 2019).

### Data analysis

Data analysis was performed using SPSS Version 27. The internal consistency of the scales was computed using Cronbach’s alpha coefficient (Cronbach, 1951) and McDonald’s omega coefficient (McDonald, 1999; Dunn et al., 2013; Hayes and Rockwood, 2019). To investigate whether the mindfulness versus control intervention had a beneficial influence on rowing-specific reinvestment scale (RSRS), Mindfulness and Flow, we conducted two groups (mindfulness, control) by two times (pre-test, post-test) ANOVAs on each dependent variable. Eta-squared, a measure of effect size, was calculated for each ANOVA. To explore within- and between-group differences we conducted *T* tests. Cohen’s *d*, a measure of effect size, was calculated for each T test (Cohen, 1992). Finally, we explored the self-reported PEQ scores to help us understand the success of the intervention and the minutes spent practicing each week.

## Results

### Demographic and descriptive statistics

A total of 82 agreed to participate but some were excluded from the analyses: 19 controls and 15 mindfulness participants did not complete the questionnaire at 6 weeks, there was no response from the participant (see Figure 1). Moreover, four missed more than one mindfulness session due to life or work commitments. Therefore, 44 rowers (32 females, 12 males), completed the study (see Table 1 for demographic data). Power calculations using GPower (Faul et al., 2007) indicated that with a sample size of 44, our study was powered at 80% to detect significant (*p* < 0.05) between-within interaction effects (*f* = 0.22, η_p_^2^ = 0.05) corresponding to a small-to-medium effect size by analysis of variance (Cohen, 1992). The descriptive statistics are in Table 2. The scale reliabilities confirmed good internal consistency for every measure, with all alpha and omega coefficients above 0.70 (Kline, 2005; Dunn et al., 2013).

**Table 2:** Descriptive statistics and within group T-tests for differences from pre-to-post for dependent variables.

			Mindfulness							Control						
			Pre		Post					Pre		Post				
	*ω*	*a*	*M*	*SD*	*M*	*SD*	*t*	*p*	*d*	*M*	*SD*	*M*	*SD*	*t*	*p*	*d*
RS-CMP	0.72	0.71	4.36	1.00	3.96	0.61	2.15^*^	0.04	0.48	5.01	0.89	4.82	0.70	1.05	0.31	0.23
RS-MSC	0.73	0.82	4.90	0.91	5.15	0.91	1.11	0.27	0.23	5.52	0.88	5.46	0.84	0.36	0.72	0.08
Flow	0.91	0.90	3.44	0.51	3.85	0.58	2.91^**^	0.008	0.61	3.95	0.49	3.88	0.72	0.72	0.48	16
Mindful Awareness	0.74	0.74	3.70	0.48	3.95	0.50	2.08	0.05	0.43	4.02	0.54	3.91	0.71	0.70	0.49	0.15
Mindful NJ	0.87	0.86	3.15	0.75	3.76	0.70	2.94^*^	0.01	0.61	3.19	0.93	3.44	0.58	1.40	0.18	0.31
Mindful Refocus	0.78	0.78	3.42	0.55	4.07	0.73	3.55^**^	0.002	0.74	3.57	0.84	3.83	0.58	1.68	0.11	0.37
Overall Training			3.13	0.46	4.35	0.71	7.93^***^	<0.001	1.65	4.38	0.71	3.80	0.83	3.56^***^	<0.001	0.78
Overall Perceived Perf	0.75	0.75	3.00	0.85	3.70	0.56	3.14^***^	<0.001	0.65	3.70	0.56	3.33	0.97	0.93	0.18	0.20
Perceived Physical	0.75	0.75	3.43	0.40	3.44	0.52	0.6	0.96	0.01	3.44	0.52	3.59	0.67	0.11	0.92	−0.02
Perceived Psychological	0.75	0.75	3.29	0.67	3.78	0.53	3.02^**^	<0.01	0.63	3.78	0.52	3.46	0.60	0.10	0.92	−0.02

RS-CMP, Rowing-Specific Conscious motor Processing; RS-MSC, Rowing-Specific Movement Self-conscious. The possible range scores for Reinvestment, Mindfulness, and Performance were 1–7. Flow was 1–5.

*** *p* ≤ 0.001;

** *p* ≤ 0.01;

* *p* ≤ 0.05.

### Rowing-specific reinvestment scale

The 2 group (mindfulness, control) by 2 time (pre-test, post-test) ANOVAs revealed main effects for group on RS-CMP and RS-MSC. Furthermore, there was a main effect for time for RS-CMP but no group × time interactions (Table 3). The control group had higher levels of RS-CMP and RS-MSC compared to the mindfulness group. RS-CMP decreased for both groups from pre to post-intervention (Table 2).

**Table 3:** Two-way ANOVA effects for dependent variables.

Variables	Time		Group		Interaction	
	*F* (1,42)	Np^2^	*F* (1,42)	Np^2^	*F* (1,42)	Np^2^
RS-CMP	5.02^*^	0.11	12.18^**^	0.23	0.54	0.01
RS-MSC	0.36	0.01	4.08^*^	0.09	1.15	0.03
Flow	2.76	0.06	1.34	0.03	6.81^*^	0.14
Mindful Awareness	0.52	0.01	1.13	0.03	3.41	0.08
Mindful NJ	9.70^**^	0.19	0.62	0.02	1.68	0.04
Mindful Refocus	14.17^***^	0.25	0.07	0.00	2.67	0.06
Overall Training	2.11	0.05	15.29^***^	0.27	32.58^***^	0.44
Overall Perceived Perf	1.83	0.04	0.30	0.01	7.63^**^	0.15
Perceived Physical	0.01	0.00	1.34	0.03	0.00	0.00
Perceived Psychological	4.69^*^	0.10	0.30	0.01	4.13^*^	0.09

RS-CMP, Rowing-Specific Conscious motor Processing; RS-MSC, Rowing-Specific Movement Self-conscious. The possible range scores for Reinvestment, Mindfulness and Performance were 1–7. Flow was 1.

*** *p* ≤ 0.001;

** *p* ≤ 0.01;

* *p* ≤ 0.05.

### Flow

The 2 group by 2 time ANOVA revealed a group by time interaction for flow but no main effects for group or time (Table 3). Follow-up T tests confirmed that the groups differed at post-intervention but not at pre-intervention (see Appendix 1). Moreover, the mindfulness group increased flow from pre-intervention to post-intervention whereas the control group did not change (Table 2).

### Mindfulness

The group by time ANOVAs revealed no group main effects or group × time interactions; however, there were time main effects for mindful refocus and mindful non-judgmental thinking (Table 3). The *t*-tests revealed that mindful non-judgmental thinking and mindful refocus increased from baseline to post-intervention in the mindfulness group but not the control group (Table 2).

### Performance

The group by time ANOVA revealed a group main effect and a group × time interaction for perceived training performance but no time main effect (Table 3). Follow-up T tests revealed that the control group exhibited higher scores at pre-intervention compared to the mindfulness group, whereas the mindfulness group exhibited higher scores at post-intervention compared to the control group (see Appendix 1). Furthermore, training scores increased in the mindfulness group but decreased in the control group from pre-intervention to post-intervention (Table 2).

The group by time ANOVA on overall perceived competitive performance revealed a group x time interaction effect but no group or time main effects (Table 3). T tests confirmed that the control group reported higher scores than the mindfulness group at pre-intervention, but the mindfulness group at exhibited higher scores post-intervention, compared to the control group (see Appendix 1). In addition, perceived performance increased from pre-test to post-test in the mindfulness group but did not change in the control group (Table 2).

The group by time ANOVA yielded a time main effect and a group × time interaction effect for perceived psychological performance but no group main effect (Table 3). Follow-tests indicated that although the groups did not differ at both pre- and post-intervention (see Appendix 1). The perceived psychological performance increased in the mindfulness group but not the control group from pre- to post-intervention (Table 2). However, there was no main group, time, or group × time interaction effects for perceived physical performance (Table 3).

### Mindfulness program evaluation

The mindfulness group reported that the intervention helped increase their ability to be in the zone (*M* = 5.79, *SD* = 0.98), reduce anxiety (*M* = 5.57, *SD* = 1.29), be focused (*M* = 5.79, *SD* = 1.06), let things go (*M* = 5.54, *SD* = 1.21), and to be aware of and cope with feelings (*M* = 5.67, *SD* = 1.24). Furthermore, they were very confident in their ability to incorporate mindfulness when rowing.

### Weekly mindfulness

The mindfulness group reported that they practiced on average 10.8 min (*SD* = 8.33), this ranged from 0 to 30 min.

## Discussion

Numerous methods have been proposed to aid performance under pressure. Mindfulness is one such method. Several putative mechanisms have been proposed to explain how mindfulness enhances performance, including increased flow (Kaufman et al., 2009; Thompson et al., 2011; Cathcart et al., 2014; Hill et al., 2020), and decreased attention on one’s movements and reinvestment (Sparks et al., 2021b). To investigate this issue further, the present study explored the effect of a sport-specific mindfulness intervention, adapted from the MMTS, on rowing-specific reinvestment, flow, mindfulness, and performance.

In line with the hypothesis, mindfulness training increased flow compared to control. This finding agrees with those of previous studies describing mindfulness interventions (Aherne et al., 2011; Scott-Hamilton et al., 2016). Nevertheless, ours is one of the shortest interventions to change dispositional flow, with the majority prescribing more than 11 h of official training (Bühlmayer et al., 2017; Noetel et al., 2017). The current finding is beneficial because athletes have demanding schedules and therefore being able to complete a mindfulness intervention in less time is preferable. Mindfulness and flow share similar characteristics, such as present moment attention and awareness (Jackson, 2016), and, therefore, it is logical that following the mindfulness training, rowers reported experiencing more flow. This is consistent with the feedback from the intervention, as athletes felt the mindfulness practice had increase their ability to stay in the present moment. The flow state has been linked with peak performance (Landhäußer and Keller, 2012) and automaticity in movement execution (Sparks et al., 2021b).

Mindfulness scores increased from pre to post in the mindfulness compared to the control group. However, contrary to our hypothesis they did not differ between each group following the 6-week intervention. Several factors may account for this null finding. First, although the intervention increased sport-specific mindfulness it may have needed to be longer to have a greater impact (Bühlmayer et al., 2017). Especially, as the control group had greater levels of mindfulness at baseline in comparison. Second, the success of an intervention in cultivating mindfulness depends on the participants’ engagement and tenacity to the practice (Zhang et al., 2016). We recommended participants complete at least 5 min practice on 5 days a week or more. However, only one participant reported completing over 30 min practice each week. Consequently, the limited amount of self-directed practice may have reduced the potential increase of mindfulness that could have resulted from the intervention.

Furthermore, in contrast to our hypothesis that mindfulness training would reduce rowing-specific reinvestment over and above the control, the intervention did not decrease conscious motor processing or movement self-consciousness compared to control. Given previous research showing that high mindfulness is associated with low RS-CMP and RS-MSC (Sparks et al., 2021b), the current null finding may be because the intervention did not increase dispositional mindfulness enough. We found that RS-CMP decreased and RS-MSC increased from pre-test to post-test in both groups. The increase in RS-MSC observed may be due to rowing training transferring during the study from initially being remote and completed in private on an erg to subsequently being back on the water in crews and with coaches observing. In accordance with trait-activation theory, whereby situational cues from specific situations activate certain traits (Geukes et al., 2013), these latter public conditions may have activated self-conscious traits in rowers, such as RS-MSC. A similar phenomenon has been observed when medical students performed a laparoscopic task—when completed in front of surgical experts, the students reported high levels of MSC, presumably because they wanted to impress their observers (Malhotra et al., 2014).

Nevertheless, consistent with our hypothesis, we found that perceived training and overall athletic performance ratings between pre-test and post-test increased more for the mindfulness group compared to the control group. Similarly, perceived ratings of psychological performance followed the same trend, which supports previous research showing that mindfulness is associated with improved team-cohesion (Baltzell et al., 2014; Piasecki, 2018), motivation (Birrer and Morgan, 2010), and concentration (Chen and Meggs, 2020). This finding highlights the versatility of mindfulness interventions over traditional psychological skills training, that may only equip athletes with tools to self-control their internal states momentarily (Birrer et al., 2012). Nevertheless, perceived psychological performance improved in both groups from pre-test to post-test. This may be explained by changes within the mesocycle—some rowers transitioned from training alone during COVID-19 lockdown isolation to training with a coach in a crew-setting when restrictions were eased. It is to be expected that receiving real-time feedback from coaches and training with a crew on the water will have improved a rower’s concentration, motivation, aggression, and team cohesion. Nevertheless, perceived physical performance did not change for either group, which may reflect the short timescale, as limited physiological changes can be expected to have occurred in 6 weeks in these experienced rowers.

Overall, the participants reported positive experiences with the intervention. The PEQ confirmed that the intervention helped athletes to be “in the zone,” reduce anxiety, augment present moment focus, let go of distractions, and be more aware of and better cope with feelings. Furthermore, after completing the intervention, they were confident that they would continue to incorporate mindfulness when rowing, both during training and competition. Overall, the feedback demonstrated that the program equipped the rowers with some of the skills needed to be successful under pressure, especially in terms of reducing anxiety and exhibiting a present moment focus, which is an important feature of flow-state (Bishop et al., 2004).

### Limitations and future considerations

There are several study limitations that need to be considered when interpreting the present findings. First, the sample size was relatively small, and there was a 50% drop out rate, and, therefore the numbers in each group may have not been large enough to detect significant changes in some variables with small effect sizes, such as performance and mindfulness. Moreover, the drop out may have resulted in selection bias, specifically for the mindfulness group, as those that did not complete the sessions, this may have been due to them perceiving the intervention as not beneficial compared to those that attended all. Future research needs to implement strategies to try and minimize attrition and drop out, such as having a reward-based system for those who complete the mindfulness practice in and outside of the official practice time. This should help keep athletes motivated to complete the full 6 weeks and the 5 days of 5–10 min of training per week (Roger-Hogan et al., 2021). Additionally, dropout rates could be reduced for the control group if they received an intervention, such as completing breathing-based relaxation training (Rooks et al., 2017). Second, there was no blinding of the participants or the principal investigator, therefore, this may have created performance bias, which could have influenced how the participants behaved and answered the measures (Probst et al., 2016). Third, there was no actual performance measure, only self-report ratings, meaning they may not be subject to social desirability bias. Nevertheless, objective performance measures could not be conducted due to COVID-19 as there were no races or erg tests organized by clubs during the season. Accordingly, future research could include kinematic measures of technical performance using telemetry. Furthermore, psychophysiology measures could also be included to support measures of reinvestment, flow, and mindfulness, as consciousness or cognitions can change over time and situations (Peifer et al., 2014). Lastly, this study demonstrated the possible successes of a sport-specific mindful intervention; therefore, it would be worth developing more sport-specific mindful interventions for different sports and investigating the impact of these.

## Conclusion

The present study provides only exploratory results as due to the high drop-out there may be a selection bias and therefore the results need to be taken with this in mind. The 6-week sport-specific mindfulness intervention demonstrated that this timescale may be long enough and effective at increasing dispositional flow. Furthermore, the intervention also increased perceived overall and psychological performance, further supporting the benefits of mindfulness. Nevertheless, 6 weeks of sport-specific mindfulness did not increase levels of mindfulness or reduce levels of reinvestment. The lack of change in these latter processes may be due to a small sample size and a lack of adherence to the home practice. Therefore, addressing the limitations, replicating the intervention and examining the possible mediators and moderators of the mindfulness-performance relationship would significantly add to the literature.

## Data availability statement

The original contributions presented in the study are included in the article/supplementary material; further inquiries can be directed to the corresponding author.

## Ethics statement

The studies involving human participants were reviewed and approved by University of Birmingham. The patients/participants provided their written informed consent to participate in this study.

## Author contributions

KS: conceptualization, formal analysis, investigation, writing, and project administration. CR: conceptualization, formal analysis, investigation, and writing. All authors contributed to the article and approved the submitted version.

## Funding

KS research is funded by the Economic and Social Research Council (grant number ES/P000711/1) and University of Birmingham Open Access Library has paid for the open access publication fees.

## Conflict of interest

The authors declare that the research was conducted in the absence of any commercial or financial relationships that could be construed as a potential conflict of interest.

## Publisher’s note

All claims expressed in this article are solely those of the authors and do not necessarily represent those of their affiliated organizations, or those of the publisher, the editors and the reviewers. Any product that may be evaluated in this article, or claim that may be made by its manufacturer, is not guaranteed or endorsed by the publisher.

## Appendix

### Appendix 1

Table 1 Difference between the groups at pre- and post-intervention.

**Table:**

	Pre-intervention			Post-intervention		
	*t*	*p*	*d*	*t*	*p*	*d*
RS-CMP	2.24	0.03	0.68	4.24	<0.001	1.28
RS-MSC	2.27	0.03	0.68	1.17	0.25	0.35
Flow	0.39	0.70	0.12	2.53	0.02	0.76
Mindful Awareness	−2.15	0.04	0.65	0.19	0.84	0.06
Mindful NJ	−0.13	0.22	0.04	1.64	0.11	0.49
Mindful Refocus	−0.73	0.47	0.22	1.21	0.23	0.36
Overall Training	5.28	< 0.001	1.59	2.34	0.02	0.71
Overall Perceived Perf	2.07	0.022	0.63	1.54	0.023	0.47
Perceived Physical	−0.85	0.40	−0.26	−0.82	0.42	0.25
Perceived Psychological	0.74	0.46	0.22	1.83	0.07	0.55
