# Supplementary material for: A rowing-specific mindfulness intervention: Effects on mindfulness, flow, reinvestment, and performance
Source: Front Psychol. 2022 Sep 7;13:871804. doi: 10.3389/fpsyg.2022.871804 (PMC9491153; doi:10.3389/fpsyg.2022.871804)
Supplement: Supplementary file 1 [file Presentation_1.pdf]

Supplementary Presentation

6-week rowers' mindfulness

Week 1

- Introductions – myself and each of the participants, sharing what they hope to get out of the MSPE – in relation to their sport and other aspects of their lives
- Confidentiality – explaining what people will be disclosing
- Rationale for Mindfulness
- Raisin/chocolate exercise
- Diaphragmatic breathing and sitting meditation – 3-minute breathing exercise
- 5-minute centering rowing exercise
- Questions in relation to this practice
- Mindfulness helps reduce the tendency to be on automatic pilot – problems with automatic pilot
- Daily home practice

Week 2

- Diaphragmatic breathing and sitting meditation – 3-minute breathing exercise
- Discussions of home practice and overcoming practice obstacles
- Mindfulness and Performance
- Rowing Body scan – re-establishing contact with the body and cultivating moment-to-moment awareness
- Discuss of Body scan
- Responding to thoughts
- Labelling exercise
- Home practice (mindful erging)

Week 3

- Diaphragmatic breathing and sitting meditation – 3-minute breathing exercise
- Discussions of home practice and overcoming practice obstacles
- Self-awareness – walking the boat exercise
- Discussion of boat exercise
- Open awareness in the boat practice
- Debrief open awareness practice
- Home practice (mindful water practice)

Week 4

- Diaphragmatic breathing and sitting meditation – 3-minute breathing exercise
- Discussions of home practice and overcoming practice obstacles
- Self-compassion in sport performance
- Imagining a difficult scene but use self-compassion cues
- Debrief difficult practice

- Home practice

Week 5

- Diaphragmatic breathing and sitting meditation – 3-minute breathing exercise
- Discussions of home practice
- Self-regulation in sport
- Value-driven performance practice
- Debrief value-driven practice
- Obstacles - Controllables versus uncontrollable
- Bullseye task
- Home practice

Week 6

- Discussions of home practice
- Pre-performance Mindfulness imagery script
- Focus circle task
- Understanding flow-state
- Home practice
